# Sensitive seismic sensors based on microwave frequency fiber interferometry in commercially deployed cables

**DOI:** 10.1038/s41598-022-18130-x

**Published:** 2022-08-17

**Authors:** Adonis Bogris, Thomas Nikas, Christos Simos, Iraklis Simos, Konstantinos Lentas, Νikolaos S. Melis, Andreas Fichtner, Daniel Bowden, Krystyna Smolinski, Charis Mesaritakis, Ioannis Chochliouros

**Affiliations:** 1 Department of Informatics and Computer Engineering, University of West Attica, Aghiou Spiridonos, 12243 Egaleo, Greece; 2 Dept. of Informatics and Telecommunications, National and Kapodistrian University of Athens, 15784 Athens, Greece; 3 Electronics & Photonics Laboratory, Dept. of Physics, University of Thessaly, 35100 Lamia, Greece; 4 Department of Electrical and Electronics Engineering, University of West Attica, Aghiou Spiridonos, 12243 Egaleo, Greece; 5 National Observatory of Athens, Institute of Geodynamics, Athens, Greece; 6 Department of Earth Sciences, ETH Zurich, Zurich, Switzerland; 7 Dept. Information and Communication Systems Engineering, Engineering School, University of the Aegean, Palama 2, 83200 Samos, Greece; 8 Hellenic Telecommunications Organization S.A. (OTE), 1, Pelika & Spartis, Maroussi, Athens, Greece

**Keywords:** Natural hazards, Imaging and sensing, Fibre optics and optical communications, Microwave photonics

## Abstract

The use of fiber infrastructures for environmental sensing is attracting global interest, as optical fibers emerge as low cost and easily accessible platforms exhibiting a large terrestrial deployment. Moreover, optical fiber networks offer the unique advantage of providing observations of submarine areas, where the sparse existence of permanent seismic instrumentation due to cost and difficulties in deployment limits the availability of high-resolution subsea information on natural hazards in both time and space. The use of optical techniques that leverage pre-existing fiber infrastructure can efficiently provide higher resolution coverage and pave the way for the identification of the detailed structure of the Earth especially on seismogenic submarine faults. The prevailing optical technique for use in earthquake detection and structural analysis is distributed acoustic sensing (DAS) which offers high spatial resolution and sensitivity, however is limited in range (< 100 km). In this work, we present a novel technique which relies on the dissemination of a stable microwave frequency along optical fibers in a closed loop configuration, thereby forming an interferometer that is sensitive to deformation. We call the proposed technique Microwave Frequency Fiber Interferometer (MFFI) and demonstrate its sensitivity to deformation induced by moderate-to-large earthquakes from either local or regional epicenters. MFFI signals are compared to signals recorded by accelerometers of the National Observatory of Athens, Institute of Geodynamics National Seismic Network and by a commercially available DAS interrogator operating in parallel at the same location. Remarkable agreement in dynamical behavior and strain rate estimation is achieved and demonstrated. Thus, MFFI emerges as a novel technique in the field of fiber seismometers offering critical advantages with respect to implementation cost, maximum range and simplicity.

Detailed imaging of Earth structure, including active rupture zones, is of paramount importance for the estimation of natural hazards^1–3^. Although significant progress has been made regarding the investigation of seismic properties and hazard of fault zones in terrestrial areas^4,5^, the structure of seismogenic submarine faults often remains poorly constrained. Furthermore, landslides and turbidity currents pose significant geohazards for marine infrastructure^6,7^. These geographic areas of interest are not easily accessible, often at a distance of hundreds of km away from the shore. Currently, the only viable solution for seismic data acquisition is the use of ocean bottom seismometers, which however poses obstacles in positioning and retrieval^8^.

During the last decade there have been many studies demonstrating that optical fiber cables in terrestrial, and most importantly in submarine installations, can operate as distributed seismometers of high accuracy providing the possibility of telemetry and continuous operation. Although optical fibers have been progressively installed since the early 1980s in order to enable broadband communication around the globe^9,10^, surprisingly, the sensitivity of optical fibers to mechanical vibrations transforms them into a potential global platform for the detection and monitoring of a wide range of geophysical and environmental effects. Exploiting such sensors around the globe enables significant applications in early warning systems and could also provide a vast amount of data for serving open science in geophysical and climate change studies. Nevertheless, massive deployment also requires both a sensitive and cost-efficient optical measurement method. The prevailing sensing technique for detecting seismic events and other environmental disturbances is distributed acoustic sensing (DAS)^11–15^. DAS is based on Rayleigh back-scattering (RBS) of light, and can detect and measure vibrations along the fiber in amplitude, frequency and phase domains^16–18^. Commercially available DAS interrogators based on phase demodulation can offer spatial resolution on the order of 1 m, distance coverage up to around 100 km at maximum and minimum detectable strain of few nanostrain and below^19,20^. DAS systems have been successfully utilized in earthquake detection and in the detailed characterization of the structure of submarine faults^21–23^, thus proving that optical fibers can provide enhanced visibility in places where human access and installation of special instrumentation is challenging. Despite its superb merits in terms of spatial resolution and sensitivity in strain measurement, DAS exhibits fundamental limitations due to its intrinsic reliance on RBS. Notably, the main drawback of DAS is that it is very sensitive to reflections caused by non-ideal connections between different fiber segments in installed deployments and generally cannot operate beyond roughly 50–100 km distances due to the low signal to noise ratio value of the back-scattered signal^20^. This constraint makes DAS quite incompatible with studies that seek to leverage long transoceanic cables for deep ocean explorations. Moreover, in order to enhance DAS reach with the use of distributed amplification, strong lasers and coding^24^, it should be preferably deployed in dark fibers^22^, meaning no other communication channels are supposed to co-propagate in the fiber under interrogation, which does not comply with the plans of telecom operators for 100% deployment of the installed fibers. Finally, DAS tools, as commercial products, are quite expensive (in the order of 100 k$) making their massive use in multiple fiber links simultaneously cost ineffective^25^.

In 2018, Marra et al. proposed the use of laser interferometry as an innovative technique for the detection of earthquakes in optical fibers^26^. Their work proved that ultra-stable laser-based interferometry is capable of detecting distant earthquakes (25–18,000 km epicentral distance with respect to the interrogated fiber link) in long fiber links (75–500 km). This technique is compatible with wavelength division multiplexing, provides remarkable sensitivity performance at optical wavelength resolution and can support long spatial ranges (> 100 km). Its main weakness is that it requires low-linewidth sub-Hz lasers which are expensive—of the same order of DAS systems in terms of cost—and complex devices and quite noisy in the low-frequency region, as a result of their sensitivity to the 1/*f*^2^ noise attributed to the random walk of laser’s phase^27,28^. This technique could indeed emerge as a strong alternative to DAS, provided that photonic integrated laser sources of ultra-low linewidth will become a mature counterpart to bulk solutions in the near future^29^. Very recently, Zhan et al. have revealed the possibility of tracking fiber deformations due to external forces by simply monitoring polarization variations in commercially deployed transoceanic links employing the already installed digital coherent transceivers^30^. The theoretical foundation of this method is presented by Mecozzi et al.^31^ and clearly shows the dependence of polarization fluctuations on the square of the local strain. This polarization sensing is a very elegant technique that is directly supported by the operating transceivers of long-haul optical communication systems. However, it is less sensitive than techniques based on phase detection^26^ and monitoring of state of polarization is almost impossible in terrestrial, “noisy” fibers due to the high sensitivity of polarization in temperature and mechanical variations caused mostly by human activity^30^.

In this work, we present in detail a new sensing technique accomplishing the detection of geophysical effects with the use of optical transmission over long distances^32^. It relies on the dissemination of a microwave frequency modulating optical carriers along a fiber link in a closed-loop configuration as depicted in Fig. 1, which we call microwave frequency fiber interferometer (MFFI). At the reception, the signal returns back to the transmitting end through the closed-loop link, and after photodetection is mixed with the transmitted microwave frequency signal in order to extract phase deviations which correspond to fiber deformations and temperature variations (Fig. 1). Microwave photonics techniques have been utilized in sensing applications in order to measure for example temperature variations^33^, strain^34^ and displacement^35^^.^ Here, for the first time, a microwave technique is proposed as an enabling platform for fiber-optic seismology, evaluated in an installed terrestrial fiber network. MFFI technique is inspired by the challenging experiments in transferring ultra-stable microwave frequency standards over “noisy” optical fiber links^36,37^. At that time, the task was to compensate for the phase noise attributed to temperature variations and mechanical vibrations affecting the transmission medium and the stability of the disseminated reference. The present work seeks to propose a technique that effectively records this phase noise and seamlessly analyzes its dynamical behavior. The use of a stable microwave frequency instead of an optical one^26^ reduces spatial accuracy (in the order of μm instead of nm^35^), however this process is far more stable—for instance the system is immune to polarization effects- and easier to control with fast sensing speed and low cost. Moreover, for the specific application where a long interferometer in the order of thousands of km is deployed, the spectral purity of the source is very important. Microwave oscillators of high spectral purity (sub-Hz frequency stability) are orders of magnitude cheaper than their laser counterparts, thus reducing significantly the overall cost of the sensing mechanism (< 5 k$) and enabling its massive deployment. The technique permits the co-propagation of the modulated optical signal with other communication wavelengths in a wavelength division multiplexing transmission (WDM) system and has a high tolerance to losses and dispersion effects, as it relies on the transmission and detection of a microwave frequency. Even for hundreds of km of transmission, a high signal to noise ratio signal can be attained at the reception end for phase comparison. In this work, we evaluate this technique using off-the-shelf low-cost components in a terrestrial, underground, 25 km-long link (50 km in the closed-loop configuration) provided by the Hellenic Telecommunications Organisation S.A. (OTE) at the area of Marousi, at the premises of OTE Academy in Attika, Greece. The system has been operating almost seamlessly since July 2021 and has successfully recorded several local and regional earthquakes with sufficient sensitivity which can be vastly improved if critical components of the overall system are optimized in a next generation of the present prototype. It is also important to state that the technique proved to be efficient in detecting earthquakes in a rather noisy terrestrial fiber spanning across densely populated areas close to Athens city center. Its efficacy in submarine environments is expected to be even higher. The time-series of MFFI are analyzed versus the signals obtained by an accelerometric station of the National Observatory of Athens, Institute of Geodynamics (NOA), operated at a nearby location and a DAS unit manufactured by Silixa Ltd which utilized the same fiber, in experiments that took place at Marousi from late September to mid-October 2021. Both comparisons reveal that MFFI provides highly correlated time-traces with those captured by the accelerometer and DAS in significant selected earthquake events from local and regional distances (> 400 km epicentral distance). Moreover, the comparison between MFFI and DAS confirmed that MFFI estimates the average strain experienced by the optical fiber as theoretically expected.

**Figure 1:**
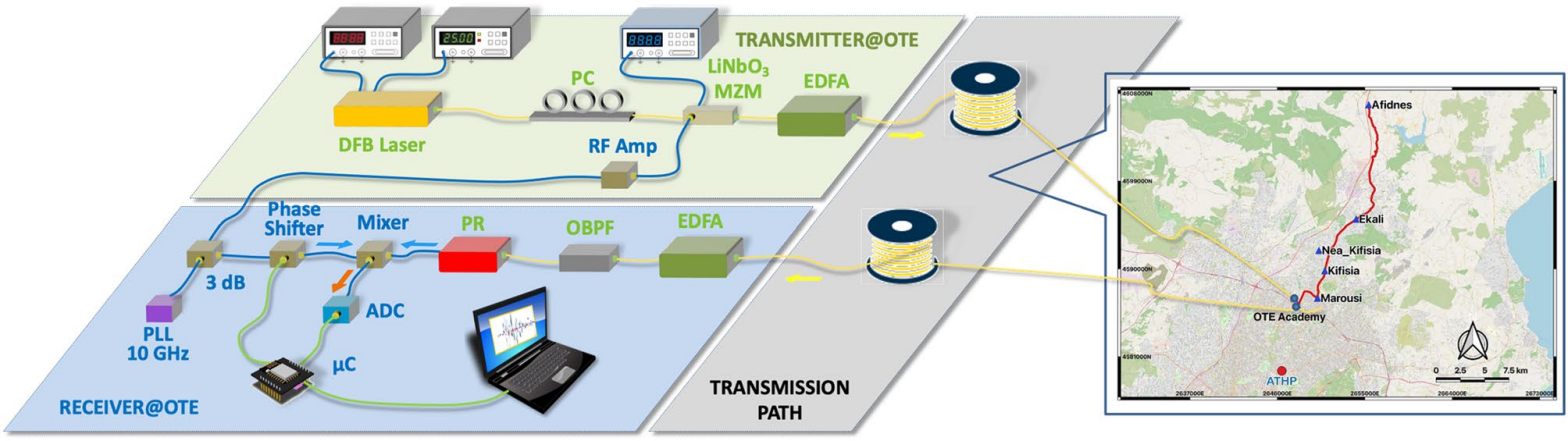
The experimental setup/conceptual scheme of MFFI: The experimental testbed was installed at OTE Academy. The transmitter consists of a distributed feedback (DFB) laser followed by a polarization controller and a Mach–Zehnder modulator of 10 GHz bandwidth, driven by a 10 GHz tone. An erbium doped fiber amplifier (EDFA) is used to boost the transmitted power. Light propagates from OTE Academy following the path to Marousi–Kifisia–Nea Kifisia–Ekali–Afidnes and vice versa (closed loop) entering the receiver’s EDFA at OTE Academy after approximately 50 km of transmission which translate to 25 dB of losses due to inefficient connections along the link that were inserted in order to emulate a longer link (125 km). The received signal, after optical amplification and proper optical filtering with the use of an optical band-pass filter (OBPF) for the reduction of amplifier’s noise is photodetected and mixed with the transmitter’s signal in order to extract the phase noise attributed to optical transmission. The baseband signal of phase noise is digitized with the use of an analog to digital converter and processed by a computer (see methods). Fiber deformations due to seismic events are imprinted on the phase noise. The map has been created using open software QGIS ver 3.16LTR (https://www.qgis.org/en/site/index.html).

**MFFI architecture** The conceptual scheme of MFFI is presented in Fig. 1. The laser is a typical 1550 nm or 1310 nm diode laser. 1550 nm is preferred due to lower losses and efficient optical amplification techniques which permit the extension of reach. The laser is externally modulated as in Fig. 1 with a microwave signal of high frequency (≥ 10 GHz). The higher the frequency, the better the phase resolution as it will be shown, however as frequency increases, the requirements of high-quality electronics scale up proportionally. The microwave frequency superimposed on the optical carrier travels along the fiber and returns back to the transmitter side using a second fiber of the same cable—thus a loopback connection is required at the end of the path. Accordingly, the signal can be amplified along the optical route using the already installed erbium doped fiber amplifiers serving WDM links. The signal is detected by a fast photodiode and then is compared with the locally generated carrier with the use of a microwave mixer. This interferometric comparison provides the phase difference between the two RF tones which is proportional to the accumulated propagation delay along the link. External disturbances on the fiber cause the modulation of the propagation delay as a combined result of variations in both refractive index and fiber length. These changes are imprinted on the phase measurements of our system. The phase difference between the detected roundtrip and the transmitted microwave signal is given by $\varphi =\frac{2\pi {f}_{RF}{n}_{g}L}{c}$ where *f*_*RF*_ is the microwave frequency of the oscillator, *n*_*g*_ is the refractive index of the fiber, *L* is the roundtrip fiber length and *c* the speed of light in vacuum (see supplementary information). The phase *φ* is stationary, as long as there is no mechanical perturbation or thermal variation along the fiber. Phase measurements can be easily transformed to strain $\varepsilon =\frac{d\varphi }{\varphi }$, where *dφ* is the accumulated variation of the stationary propagation phase *φ* of fiber as a result of a mechanical deformation, to strain rate ($\frac{d\varepsilon }{dt}$) for comparison in terms of spectro-temporal properties to what seismometers (proportional to $\frac{d\varepsilon }{dt}$) or accelerometers (proportional to ${d}^{2}\varepsilon /d{t}^{2}$) measure. The effect of temperature variations in *dφ* is eliminated with post processing of the signal (see supplementary information). From the above analysis, we can easily deduce that the average strain can be straightforwardly calculated as follows:

1 $$\varepsilon =\frac{cd\varphi }{2\pi {n}_{g}\xi {f}_{RF}L}$$

The parameter *ξ* is the strain coefficient of the optical fiber due to the photoelastic effect^38^ and equals to 0.78 approximately.

In the experimental setup of Fig. 1, the critical part is the receiver which is explicitly described in Methods. It must be noted that two erbium doped fiber amplifiers (EDFAs) have been used at the transmitter as a booster and at the receiver as a pre-amplifier in order to improve signal to noise ratio. Both transmitter and receiver reside at OTE academy and the optical link traverses Marousi–Kifisia–Nea Kifisia–Ekali–Afidnes and vice versa (closed loop) forming a 50 km long link with total losses approaching 25 dB due to inefficient connections at intermediate points that were intentionally introduced so as to mimic an almost 125 km long link.

## Results

### Comparison of MFFI with accelerometers

**Figure 2:**
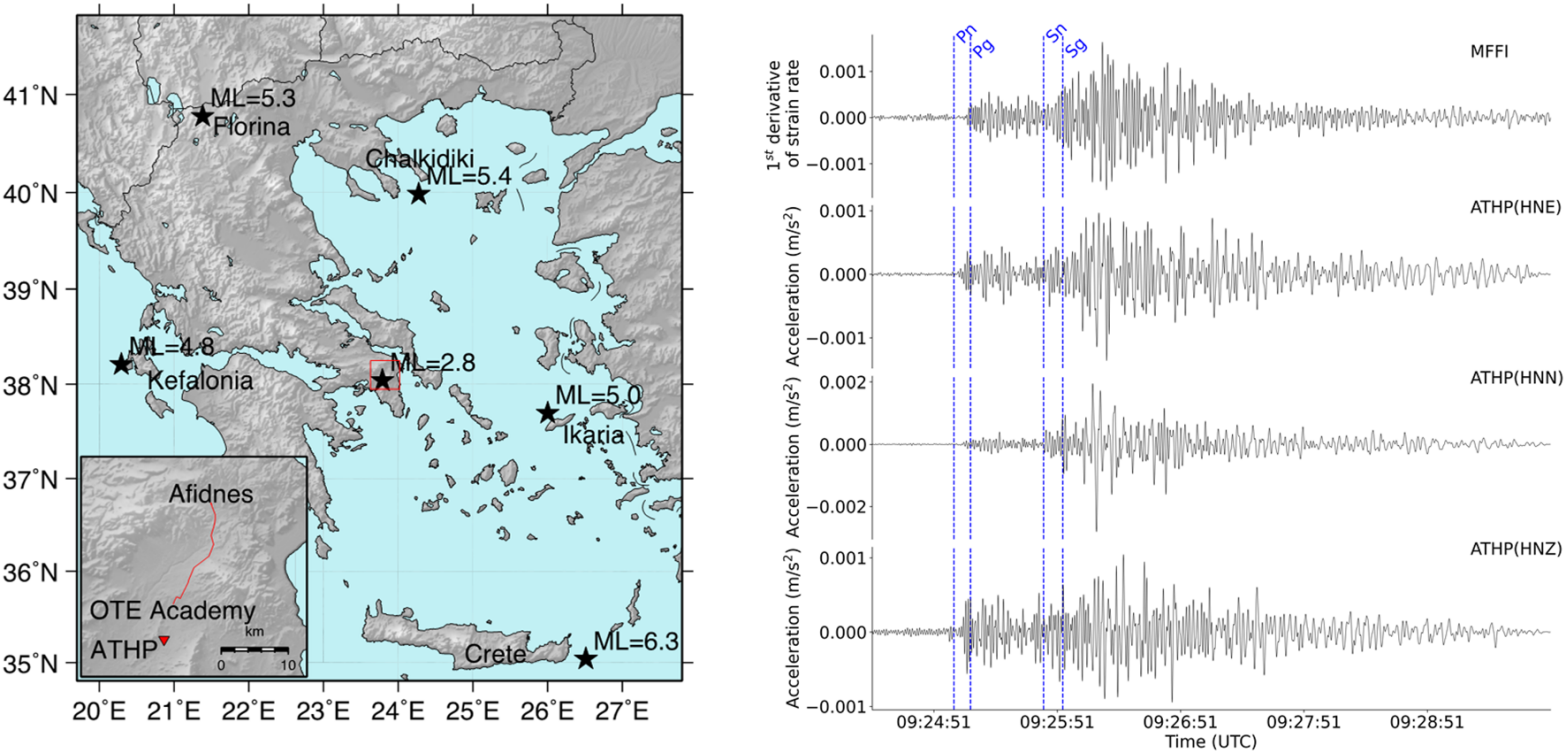
MFFI comparison to ATHP accelerometer. (Left) A map of Greece showing in a red box the location of MFFI system and ATHP accelerometric station (the inset magnifies the area contained in the rectangular frame) and the epicenters with black stars of earthquakes selected for analysis in the present study. (Right) Comparison of the time series collected by MFFI and ATHP regarding the earthquake taking place east of Crete Island (October 12, 2021, 09:24:03 UTC, Crete (ML = 6.3)). Pg/Sg, Pn/Sn theoretical arrival times are marked with blue dotted lines for reference. Map on the left has been created using open software GMT ver 5.4 (https://www.generic-mapping-tools.org/).

The installed MFFI has been evaluated as an accelerometer through its comparison with conventional accelerometers. We used three-component waveform data recorded at ATHP (Athens—Neo Psichiko), the nearest to the optical link accelerometric station of the National Accelerographic Network of the Institute of Geodynamics, National Observatory of Athens. We have collected recordings of selected earthquakes taking place during the testing period of the system (from July 2021 to February 2022). The epicentral locations (Crete, Marousi, Ikaria, Kefalonia, Florina, Chalkidiki) of diverse selected seismic events for analysis are depicted on the map of Fig. 2, (left side), while in the inset of the same figure we can see the locations of the MFFI and ATHP. The comparison between MFFI and ATHP data for all different epicenters (black filled stars in Fig. 2), are included in the supplementary information. Figure 2, right, compares the three-component ground acceleration time-series at ATHP station (HN denotes the high sampling rate, 200 samples/sec for a three-component accelerometer sensor, with Z the vertical component and E, N the E–W and N–S horizontal components, respectively) against the first derivative of strain rate measured by MFFI for the 12 October 2021, ML = 6.3, Crete earthquake. Theoretical arrival times of Pn/Sn and Pg/Sg based on ak135 velocity model are shown in blue for reference^39^. Τhe ak135 model is an 1D, isotropic, radial velocity model derived from empirical travel times with the purpose to fit a wide range of seismic phases, such as direct phases, diffracted phases, core reflections, surface reflections and core phases. As a result, it is very effective and it is widely used for global event location and phase associations. It has to be noted that, given the shallow depth of the Mw = 6.3, Crete earthquake (7.6 km), Pn/Sn seismic phases represent Primary wave (P) and Secondary wave (S) phases respectively, bottoming on the uppermost mantle, whilst Pg/Sg are P and S waves bottoming on the upper crust^40^.The data is bandpass filtered from 0.1 Hz to 1.0 Hz and the MFFI time-series are normalized in the amplitude so as to transform the derivative of strain rate to acceleration. Although an accurate conversion of strain to ground motion is feasible if wave’s apparent phase velocity is known^41^, in this work, for the sake of simplicity, we apply a scaling factor that normalizes both sources of data with respect to their mean power. An agreement between strong motion data and MFFI P and S onsets is observed, especially on the horizontal components. Notably, the two signals appear to be in phase and the P/S phase identification criteria (difference in amplitude and frequency in the two horizontal components) seem to hold for the MFFI signal too, which reveals the potential of MFFI to constitute a new tool for body wave arrival time picking/recognition that could be embedded in early warning systems.

**Figure 3:**
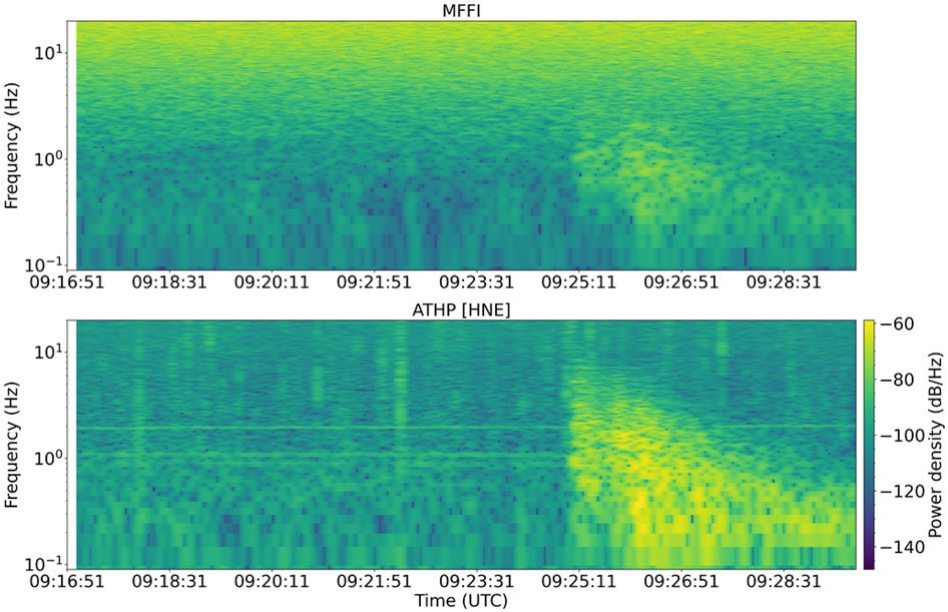
Spectrograms: The spectrogram generated by MFFI time-series (first derivative of strain rate) is compared to the spectrogram of the ΗΝΕ components of ATHP acceleration recordings. Similar dynamics are deduced, despite the high noise impairing MFFI spectral components above 5 Hz, attributed to low-cost solutions employed and the high noise imparing the terrestrial link.

In Fig. 3, the unfiltered spectrograms for the same event are depicted (power density in dB/Hz). Spectrograms compare the HNE component of ground acceleration recordings at the ATHP station against the derivative of the strain rate time-series captured by MFFI for the 12 October 2021, ML = 6.3, Crete earthquake. A substantial increase in the observed energy from 0.1 Hz up to 3 Hz coincides with the arrival times of P and S waves, excited by the Crete earthquake. Notably, the first P wave arrival at ATHP station is observed at 09:25:02 UTC, whilst the first S arrival is observed approximately 50 s later. This is evident in Fig. 3, where HNE horizontal components show peak energy levels, below 0.5 Hz, only after 09:26:00 UTC approximately. Beyond 09:27:00 UTC high frequency body wave arrivals have attenuated substantially, while surface waves having longer-periods (10–20 s) dominate the seismograms. Similar characteristics are observed on the spectrogram produced based on MFFI time-series, nonetheless, the signal-to-noise ratio is significantly lower than that of the strong motion data. The very high level of frequency noise above 5 Hz across the entire time range is due to our choice to demonstrate the efficacy of the system solely relying on low-cost components and due to the increased noise characterizing terrestrial links as a result of human activity. This noise is amplified at higher frequencies (> 5 Hz) as an effect of second-order differentiation. Techniques for further optimization of the system are described in methods.

### Comparison of MFFI with DAS

**Figure 4:**
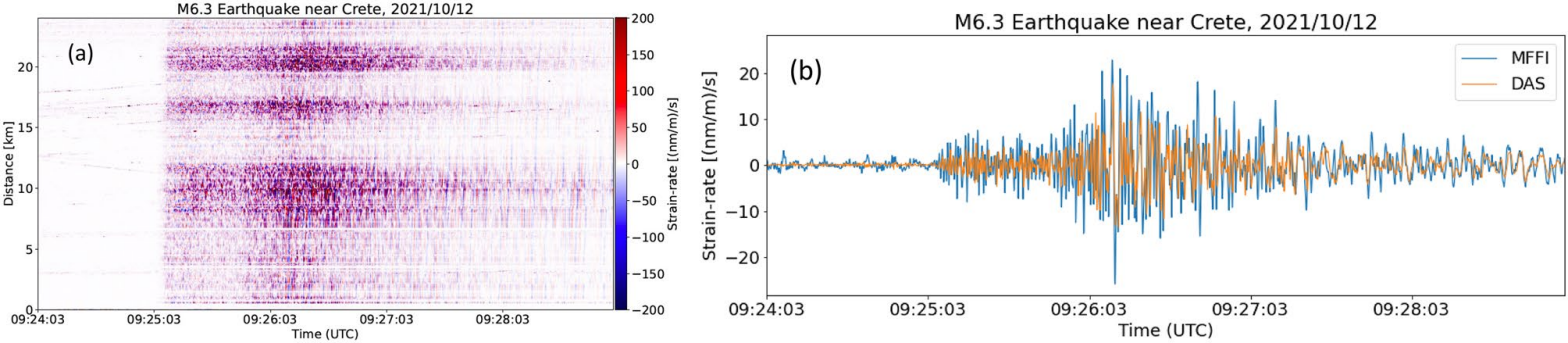
MFFI comparison to DAS interrogator. (**a**) DAS records corresponding to the earthquake taking place east of Crete island [October 12, 2021, 09:24:03 UTC, Crete (ML = 6.3)], (**b**) MFFI strain rate vs. average DAS strain over spatial dimension. The agreement in the estimation of strain rate between the two systems is obvious.

From late September to mid-October 2021, we had the opportunity to compare MFFI with a commercially available, iDAS interrogator produced by Silixa ltd. The two systems had been operating simultaneously on the same fiber path for three weeks. For this experiment we used a 2 m spatial resolution, 10 m gauge length, and 400 Hz recording rate in DAS. For these parameters, the maximum reach of iDAS was close to 25 km for the specific link characterized by excess losses. We carried out the comparison between DAS and MFFI relying on the data of Crete earthquake (12 October 2021, ML = 6.3 Crete earthquake). In Fig. 4a, we see data produced by DAS that depicts strain rate as a function of time and location in the fiber with a spatial resolution of 2 m. The manifestation of the earthquake on the evolution of strain rate is evident. What is important to observe is that strain rate is not uniformly recorded along the link. On the contrary there are certain parts of the fiber that exhibit stronger strain rate than others. In order to compare the two systems, we calculated the average strain rate of all local sensors (channels) recorded by DAS with a spatial separation of 2 m (see Fig. 4a) (see methods). Since both spatial resolution and gauge length are shorter than the wavelength of the seismic wave, this allows us to ignore their effects and to approximate the integral along the fiber by a simple sum. MFFI strain rate and DAS average strain rate are depicted in Fig. 4b after filtering the signals from 0.1 to 1.5 Hz. It becomes evident that MFFI’s measurement coincides with the average strain experienced by the optical fiber link as expected according to Eq. (1). The two signals have slightly different amplitudes because the MFFI signal is more susceptible to noise mostly related to the analog to digital converter (ADC) performance (see supplementary information). Therefore, MFFI technique provides spatially averaged strain rates measurements that are also provided by a high maturity, commercially available DAS system.

## Discussion

MFFI has been successfully benchmarked in real life conditions and it has been proven capable of detecting a wide variety of seismic events with epicenter distances ranging from a few km (Marousi ML = 2.8) to a few hundreds of km (Crete ML = 6.3). The exhaustive testing of MFFI in a commercially deployed network that resides in a densely populated area of Athens provided realistic limits on its performance. The sensitivity of the system depends on the optical signal to noise ratio (OSNR) of the received signal (OSNR = 43 dB in our setup), the RF frequency, the stability of the transmitter, the resolution of the ADC as well on the electronic noise at the receiver end. It is also dependent on the link noise which is a superposition of temperature fluctuations and acoustic noise caused by human activity (car traffic, construction, subway, etc.). Despite the fact that the terrestrial link is routed close to highways and within densely populated areas with a depth from the surface varying from 40 cm to 2.5 m, the MFFI has been proven very robust and selective in identifying seismic events which are polluted by link noise (see supplementary information about link noise). Its efficacy in noise-free submarine environments is expected to be orders of magnitude larger as has been proved for equivalent techniques^26^. In the present implementation that utilizes low-cost, off-the-shelf electronic components, the sensitivity is mainly limited by ADC quantization noise and is estimated to be 0.62 mrad. The overall cost of a pluggable optoelectronic device operating as MFFI in a separate wavelength in a long-haul transmission system, thus re-using all link resources such as in-line optical amplifiers, is estimated to be less than 1 k$, which is almost two orders of magnitude lower than that of commercially available DAS systems. Chromatic dispersion effects could constitute a barrier at long distances (> 200 km) due to power fading effects which however can be mitigated using various techniques (see supplementary information as well)^42–44^. Further improvement could be achieved by increasing the RF modulation frequency and utilizing a higher resolution ADC to lower the quantization noise. Increase of RF modulation frequency can be accomplished with the use of carrier suppression^44^ or higher-order harmonic generation by means of optical modulation^45^. Our implementation that relies on low-cost off-the-shelf components can detect optical path variations in the order of Δ*L* ~ 2.5 μm. We envisage that this can be reduced by more than an order of magnitude with the use of a high-performance ADC (24 bit resolution, 1 kHz sampling rate) and with a factor of four if the microwave frequency is increased to 40 GHz. State of the art innovations in integrated microwave photonics^46,47^ could potentially enable the preparation of spectrally pure mm-wave carriers approaching 100 GHz, that can be detected with the use of high performance 100 GHz photodetectors^48,49^. Thus, keeping in mind that 100 GHz components will be soon available for telecom applications, MFFI prototypes could potentially provide sensitivity at unprecedented levels. Even with the use of off-the-shelf and mature 10–20 GHz optoelectronic components, satisfactory sensitivity can be attained at low cost and in real-time which is of high importance for the development of early warning systems. On the contrary, techniques relying on extracting events related to environmental effects by processing the huge amount of data offered by digital coherent receivers^30,50^ operating in the multi GSa/sec time scale require unparalleled processing power to offer real-time identification of critical events such as tsunamis, earthquakes, etc. A massive production of high performance MFFI prototypes offering real-time event detection at minimum cost could be achieved, thus paving the way for mid-term installation of MFFI tools in almost every fiber link of interest worldwide. Thus, our results in a rather noisy fiber located in a crowded area prove that MFFI could emerge as a key enabling technology for the widespread evolution of fiber optic seismology. MFFI can also provide the possibility of a better localization of fiber deformations and be converted to distributed strain meters. The simplest solution is to use two MFFI systems positioned at the two ends of the link. Through cross-correlating their time traces corresponding to counter-propagating waves in a periodic basis, one can localize perturbations of the link^26,51^. The spatial resolution depends on the integration time and sampling rate at each side^51^ and could be in the order of hundreds of meters or even less which is adequate for earthquake detection as wavelengths related to earthquakes are on the order of several hundred meters or several kilometers. Beyond that straightforward approach, continuum mechanics analysis of the relation between optical phase changes and the strain tensor reveal that the sensitivity of a fiber segment to deformation is proportional to the local fiber curvature^52^. This implies that strongly curved segments, such as tight loops, effectively act as individual sensors that contribute large phase measurements $$\varphi (t)$$ at distinct times when a wavefront reaches the segment. Consequently, a time-dependent analysis of $$\varphi (t)$$ may effectively mimic a distributed system of strongly curved fiber segments^53^. This, in turn, opens new perspectives for seismic tomography and earthquake location in remote regions where dense arrays of conventional seismometers are not available and may convert a single MFFI interrogator to a distributed measurement engine.

## Methods

### Experimental setup

At the transmitter end, a distributed feedback laser (DFB) laser is connected with a polarization controller and a LiNbO_3_ modulator that imprints the microwave 10 GHz carrier on the optical carrier. The polarization controller is used in order to maximize the optical modulation depth at the output of the modulator. A combination of DFB laser with integrated electro-absorption modulator could provide higher stability of the transmission system, much lower transceiver cost and shall be considered in future implementations. The signal is amplified up to 6 dBm with the use of erbium doped fiber amplifier (EDFA) booster (Amonics, AEDFA-23-B-FA) and launched into the 50 km long link. For such an optical power level, fiber nonlinearities are not triggered. The signal arriving at Afidnes is loopbacked and propagates in a separate fiber of the same bundle so as to enter the receiver. In a commercially deployed WDM network, loopback connection can be performed without disturbing co-propagating channels with the use of WDM demultiplexers and multiplexers. At the receiver end, the propagated optical signal is amplified by a second EDFA (Amonics, AEDFA-23-B-FA), then filtered with an OBPF of 0.5 nm bandwidth for out-of-band noise reduction and finally detected by a 10 GHz bandwidth photodiode. The resulting electrical signal is sent to the RF port of a microwave mixer. The LO port of the mixer is driven by a phase-controlled replica of the 10 GHz signal that is used for the modulation of the transmitter signal. The phase control is performed by a microwave phase shifter HMC642ALC5 with a resolution of 6 bit (5.625°) and secures that the phase difference between the signal at the LO port and the received signal remains around π/2 to ensure maximum slope *ΔV*/*Δφ* where *ΔV* corresponds to voltage variation caused by a phase variation *Δφ*. This enhances sensitivity and prevents signal clipping after the DC amplification that follows. The mixer’s output is sent to a 50 Hz bandwidth, 30 dB DC coupled amplifier which removes the high frequencies of the signal and adjusts its DC voltage level for the ADC. Sampling is performed at a rate of 100 Hz by means of the 10-bit embedded analog to digital converter (ADC) of a low-cost micro-controller (Arduino Uno), which transfers the collected data to the serial port of a computer. Intentionally, we used such a low-cost system at the final processing in order to acquire remote control of the final block in a straightforward manner and evaluate MFFI performance with very low-cost electronics and in real-time, a feature that can not be straightforwardly provided by other optical sensing techniques^26,30^. Besides sampling, the micro-controller is also responsible for the control of the phase shifter. The recorded signal is proportional to the phase difference *φ* accumulated during the propagation in the fiber and carries the signature of thermal variations and mechanical vibrations that disturb the optical fiber (see supplementary information). The recorded signal is then digitally processed for rejection of both high frequency noise and very low frequency thermal effects and finally converted to strain and strain rate based on (1).

The RF oscillator is based on the HMC769 fractional-N Phase locked loop (PLL) with integrated VCO @ 9.05—10.15 GHz. The oscillator frequency is set to exactly 10 GHz with a reference frequency of 50 MHz and the loop bandwidth is set to 100 kHz. In this way, the PLL is configured as integer –N frequency synthesizer, avoiding the fractional spurious products which could impair the phase noise of the round-trip path. Taking into account that the round-trip time is 50 km/2 × 10^8^ = 0.25 ms, the fiber loop bandwidth is 4 kHz and within this bandwidth the oscillator induced phase noise of the transmitted and received microwave signal is correlated and thus rejected. We can estimate that the oscillator contribution to the total uncorrelated phase noise of the system is in the order of -110 dBc/Hz for the frequency range 4–100 kHz, well outside the frequency range of interest for earthquake detection. If one wants to extend the range to hundreds of km, the lower frequency of the oscillator uncorrelated noise shall be reduced and fall within the frequency band of interest, so higher spectral purity microwave oscillators should be employed at a reasonable cost.

### ATHP

We used three-component waveform data recorded at ATHP accelerometric station (Athens—Neo Psichiko), the nearest to the optical link provided by OTE. The station belongs to the National Accelerographic Network of the Institute of Geodynamics, National Observatory of Athens (HL, National Observatory of Athens, 1997)^54^. The station is installed at the basement of a three-floor private building, built from reinforced concrete, founded on weathered sandstone, and it is equipped with a Güralp CMG-5TDE accelerometer, consisting of a force-feedback broadband strong motion accelerometer sensor, together with a 24-bit digitizer telemetered in real time to the NOA seismicity recording center in Thissio, Athens. The sensor is oriented at N20°E in installation, which makes the N-S component parallel to the optical link main direction, and the E-W component perpendicular to it. The instrument shows a dynamic range of over 130 dB, and a flat response from DC up to 100 Hz (sampling rate at 200 Hz), which makes it ideal for the recording and study of strong earthquakes in both local and regional epicentral distances.

### Comparison between DAS and MFFI data

Most DAS interrogators measure optical phase changes and transform them into strain. Based on the literature^20,31^, the relation between strain and changes in phase is the following:

2 $$d\varphi =\frac{2\pi {n}_{g}L\xi \varepsilon }{\lambda }$$

where *n*_*g*_ is the refractive index, *L* is the length (double transit of gauge length when DAS is the strain-meter), *ξ* is a scale factor due to photo-elastic effect, *λ* is the wavelength of light in the free space (1550 nm in our case). Gauge length in typical DAS interrogators is in the order of a few meters. Therefore, strain is indirectly measured through the equation

3 $$\varepsilon =\frac{\lambda d\varphi }{4\pi n_gG\xi }$$

where *G* is the gauge length employed by the DAS system. The minimum value of *dφ* that can be measured determines the strain sensitivity of the system. MFFI measures the overall phase change experienced by a sinusoidal carrier superimposed on an optical carrier along the entire link. Therefore, the phase change that we measure is

4 $$\varphi =\frac{2\pi {f}_{RF}{n}_{g}L}{c}$$

Taking into account the photo-elastic effect here as well, then phase changes experienced by the microwave frequency caused by diverse external perturbations and their relation to strain can be provided by the following equation

5 $$d\varphi =\frac{2\pi {f}_{RF}{n}_{g}L}{c}\xi \varepsilon$$

Therefore, strain is calculated as follows:

6 $$\varepsilon =\frac{cd\varphi }{2\pi {n}_{g}\xi {f}_{RF}L}$$

MFFI measures the overall strain along the fiber, therefore it could be compared with DAS if we average all strain components measured at all spatial locations of DAS distributed measurement (integration of (3) over all distributed data and division by the number of spatial channels). This procedure has been followed and the resulting time-series is depicted in Fig. 4b, which proved the correctness of the approach.

## Supplementary Information

Supplementary Information.

## Data Availability

The datasets generated and/or analysed during the current study are available in: https://pithos.okeanos.grnet.gr/public/lBVM2xRXA86Ca8fL5GkD44.
