# Supplementary material for: Sensitive seismic sensors based on microwave frequency fiber interferometry in commercially deployed cables
Source: Sci Rep. 2022 Aug 17;12:14000. doi: 10.1038/s41598-022-18130-x (PMC9386022; doi:10.1038/s41598-022-18130-x)
Supplement: Supplementary file 1 — Supplementary Information. [file 41598_2022_18130_MOESM1_ESM.pdf]

Sensitive Seismic Sensors Based on Microwave Frequency Fiber Interferometry in Commercially Deployed Cables: Supplementary material

Adonis Bogris[1], Thomas Nikas[2], Christos Simos[3], Iraklis Simos[4], Konstantinos Lentas[5], Nikolaos S. Melis[5], Andreas Fichtner[6], Daniel Bowden[6], Krystyna Smolinski[6], Charis Mesaritakis[7], Ioannis Chochliouros[8]

1. Dept. of Informatics and Computer Engineering, University of West Attica, Aghiou Spiridonos, 12243, Egaleo, Greece
2. Dept. of Informatics and Telecommunications, National and Kapodistrian University of Athens, Athens, GR-15784, Greece
3. Electronics & Photonics Laboratory, Dept. of Physics, University of Thessaly, 35100 Lamia, Greece
4. Department of Electrical and Electronics Engineering, University of West Attica, Aghiou Spiridonos, 12243, Egaleo, Greece
5. National Observatory of Athens, Institute of Geodynamics, Greece
6. Department of Earth Sciences, ETH Zurich, Switzerland
7. Dept. Information and Communication Systems Engineering, Engineering School, University of the Aegean, Palama 2, 83200, Samos, Greece
8. Hellenic Telecommunications Organization S.A. (OTE), 1, Pelika & Spartis, Maroussi, Athens, Greece

This document provides supplementary information to "Sensitive Seismic Sensors Based on Microwave Frequency Fiber Interferometers in Commercially Deployed Cables", including details on the theoretical analysis of the interferometer, processing of raw phase data, seismic data of various selected recorded earthquakes, a first analysis of the phase noise of the link and the individual building blocks/stages of the system and propositions that could transform MFFI to a distributed earthquake sensor.

A. Theoretical analysis of the interferometer

Figure 1 shows the MFFI in a conceptual form. One can easily see that this is about a Mach-Zehnder delay interferometer where the two branches have a path difference of L_{rtr} where L_{rtr} is the total length of the fiber that is interrogated and it is a round-trip, closed-loop path starting and ending at the same point.

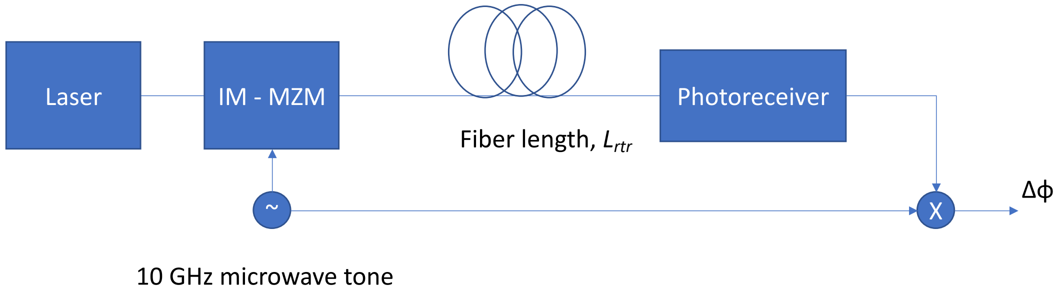

Fig. 1: Conceptual representation of the microwave frequency fiber interferometer.

The only difference with a typical Mach-Zehnder is that the phase detection is accomplished after photodetection in the RF domain as depicted above and will explained mathematically in the next paragraphs.

The modulation signal V_M applied on a Mach Zehnder modulator (MZM) is a sinusoidal voltage with angular frequency ω_{RF} and amplitude V_m superimposed on a DC voltage V_B: $V_M=V_B+V_m\sin(\omega_{RF}t)$ The output field as a function of the input voltage is given by[1]:

$$E_{out} = E_{in}\sqrt{A}\sqrt{\frac{1}{2}\{1 + BJ_0(C) + 2B\sum_{n=1}^{\infty}J_{2n}(C)\cos(2n\omega_{RF}t) - 2D\sum_{n=1}^{\infty}J_{2n-1}(C)\sin(2(n-1)\omega_{RF}t)\}}(1)$$

Where A is the optical insertion loss, $B=\cos(\pi V_B/V_\pi)$, $C=\pi V_m/V_\pi$, $D=\sin(\pi V_B/V_\pi)$ and V_π is the voltage required to change the phase of the modulated MZM arm by π, $J_n(x)$ is the n-th order Bessel function of the first kind.

Depending on C, D values, one can either promote odd or even harmonics and preserve or eliminate the DC component. The latter – if removed – will make the system immune to dispersion as it will be explained later on.

If C is properly selected, then without loss of generality, we may deduce that the output field is mostly carrying the first harmonic ω_{RF} of the sinusoidal signal.

Thus, the output of the modulator is the following:

$$E_{out} = E_{in}\sqrt{A[1 + m \cdot \cos(\omega_{RF}t + \varphi_{noise}(t))]} \qquad (2)$$

Where $E_{in} = \sqrt{P}e^{-i(\omega_{opt}t+\varphi_{opt})}$ is the optical carrier of power P, optical angular frequency ω_{opt} and optical phase φ_{opt}.

The term m is the modulation depth, φ_{noise} is the phase noise of the microwave source.

After transmission, and ignoring the chromatic dispersion effects at this point, the signal will acquire a phase shift in the microwave domain that is equal to $\varphi_{RF}=\omega_{RF}T_{prop}$ and a phase shift in the optical domain that is equal to $\varphi_{opt}=\omega_{opt}T_{prop}$, where $T_{prop}=L_{rtr}/v_g$

T_{prop} is the time required for a round-trip propagation (from the transmitter back to the receiver) which depends on the round trip distance L_{rtr} and the group velocity $v_g=c/n_g$. The signal will also experience losses equal to $A_{fiber}=\exp(aL_{rtr})$ and random polarization fluctuations.

If the signal is directly detected on a photodiode, then the photodiode will only reproduce the microwave power envelope of the modulated optical carrier, that is

$$I_{PD} = RPA_{fiber}A \cdot m\cos(\omega_{RF}t + \varphi_{noise}(t - T_{prop}) - \omega_{RF}T_{prop}) \qquad (3)$$

[1] Nikas, T., Bogris, A., & Syvridis, D. (2017). Double sideband suppressed carrier modulation for stable fiber delivery of radio frequency standards. Optics Communications, 382, 182-185.

Where I_{PD} is the photocurrent, after removing the DC component, and R is the responsivity of the photodiode. The photodiode output is sent to a microwave mixer and is combined with the local RF carrier that modulates the MZM. The output of the mixer will be the following:

$$M_{out} = V\cos\left[\omega_{RF}t + \varphi_{noise}\left(t - T_{prop}\right) - \omega_{RF}T_{prop}\right]\cos\left(\omega_{RF}t + \varphi_{noise}(t) + \varphi_{shifter}\right) \quad (4)$$

Using the well-known formula $\cos(a)\cos(b) = \frac{\cos(a-b)+\cos(a+b)}{2}$ we can easily deduce that the term of interest that remains after low pass filtering the output of the mixer is the term of cos(a-b), hence:

$$V_{out} = V\cos\left(\omega_{RF}T_{prop} - \varphi_{noise}\left(t - T_{prop}\right) + \varphi_{noise}(t) + \varphi_{shifter}\right) \quad (5)$$

If the microwave source has a high coherence length (small linewidth), then one may safely assume that $\varphi_{noise}\left(t - T_{prop}\right) \approx \varphi_{noise}(t)$. This is easily obtained for microwave frequencies of high spectral purity that can preserve their coherence for distances > 5000 km. Using for instance the well-known formula of coherence length $L_{coh} = \frac{v_g}{\pi \Delta f}$, where Δf is the linewidth of the microwave source and assuming that its value is close to 1 Hz or even below (actually tens of mHz for a high stability 10 GHz carrier), then L_{coh} > 63.000 km which proves that the system is immune to source phase noise even for transoceanic links whose length is usually below 15.000 km in the round-trip.

Therefore the output signal is:

$$V_{out} = V\cos\left(\omega_{RF}T_{prop} + \varphi_{shifter}\right) \quad (6)$$

The term $\omega_{RF}T_{prop}$ is the term $\varphi = \frac{2\pi f_{RF} n_g L}{c}$ appearing in eq. (4) of the original manuscript.

The signal is imprinted on T_{prop} which is disturbed by seismic events as explained in the manuscript. The amplitude V depends on the total amplification of the detected signal, both in RF (prior to the mixer) and low frequency regime and the RF power injection to the Local Oscillator port of the microwave mixer.

The phase shifter is continuously controlled so as to enhance the resolution of the measurement operating the system close to the linear regime of the cosine function. We note that T_{prop} is composed of a static T_{propS} and dynamic T_{propD} component, $T_{prop}=T_{propS}+T_{propD}$. The varying T_{propD} term is a function of both low frequency and higher magnitude thermal effects and higher frequency lower magnitude strain variations, the latter being the targeted signal, $T_{propD}=T_{propDTH}+T_{propDST}$. So, we can express $T_{prop}=T_{propS}+T_{propDTH}+T_{propDST}$ and thus:

$$V_{out} = V\cos\left[\omega_{RF}\left(T_{propS} + T_{propDTH} + T_{propDST}\right) + \varphi_{shifter}\right] = V\cos\left[\omega_{RF}\left(T_{propS} + T_{propDTH}\right) + \varphi_{shifter} + \omega_{RF}\left(T_{propDST}\right)\right] \quad (7)$$

In order to effectively track $\omega_{RF}T_{propDST}$ we continuously adjust the phase of $\varphi_{shifter}$ to cancel the slow thermal phase drift and obtain $\omega_{RF}\left(T_{propS} + T_{propDTH}\right) + \varphi_{shifter} = 2k\pi - \pi/2$. Then, for small $\omega_{RF}T_{propDST}$ we have:

$$V_{out} = V\cos\left(\omega_{RF}T_{propDST} - \pi/2\right) \approx V\omega_{RF}T_{propDST} \quad (8)$$

Therefore, the phase shifter functionality achieves to convert linearly V_{out} to $\omega_{RF}T_{prop}$

Photo-elastic effect

The photoelastic effect relates the change of refractive index to the mechanical strain, described by the two strain-optic coefficients p_{11} and p_{12}. By measuring the phase change of light propagating through the fiber due to a longitudinal strain, we can determine the individual values of the two coefficients p_{11} and p_{12}. Using first-order theory of elasticity and the photoelastic effect, and assuming that the fiber is elastic and mechanically homogeneous, the phase change $\Delta\varphi$ induced
by an elongation ΔL is given by[2]:

$$\Delta\varphi = \frac{\omega_{RF}}{c}\left[n_g \Delta L + \Delta n_g L\right] = \frac{\omega_{RF}}{c} n_g \Delta L \left\{1 - \frac{n^2}{2}[p_{12} - v(p_{11} + p_{12})]\right\} \tag{9}$$

Δn_g is the refractive index change and v is the Poisson's ratio of the fiber material. The parameter $\xi = 1 - \frac{n^2}{2}[p_{12} - v(p_{11} + p_{12})]$ is the strain coefficient of fiber due to the photoelastic effect and is approximately equal to 0.78[3].

Dispersion effects

Chromatic dispersion is one of the most significant deteriorating effects in fiber transmission that can cause temporal broadening and power fading. A microwave frequency can be superimposed on an optical carrier in many different ways as depicted in fig. 2. When both sidebands of the microwave frequencies are transmitted as in fig. 2a then the two sidebands acquire different phases due to dispersion. When they interact on the photodiode, the different phases result in power fading[4]. The expression providing how power fading evolves with respect to transmission distance L, second-order dispersion parameter β_2 and ω_{RF} is the following

$$P_{out} = P_{in} \cos\left(\frac{1}{2}\beta_2 \omega_{RF}^2 L\right) \tag{10}$$

Using this simple formula, one can easily find how optical power fading evolves as a function of RF frequency for a given dispersion value (β_2=-21 ps^2/km in Single Mode Fibers) and L=2500 km and L=500 km.

[2] Bertholds, Axel, and Rene Dandliker. "Determination of the individual strain-optic coefficients in single-mode optical fibres." Journal of lightwave technology 6.1 (1988): 17-20.

[3] Giallorenzi T G et al "Optical fiber sensor technology" IEEE J. Quantum Electronics, v18 626-65, 1982

[4] Cui, Y., Xu, K., Dai, J., Sun, X., Dai, Y., Ji, Y., & Lin, J. (2012). Overcoming chromatic-dispersion-induced power fading in ROF links employing parallel modulators. IEEE Photonics Technology Letters, 24(14), 1173-1175.

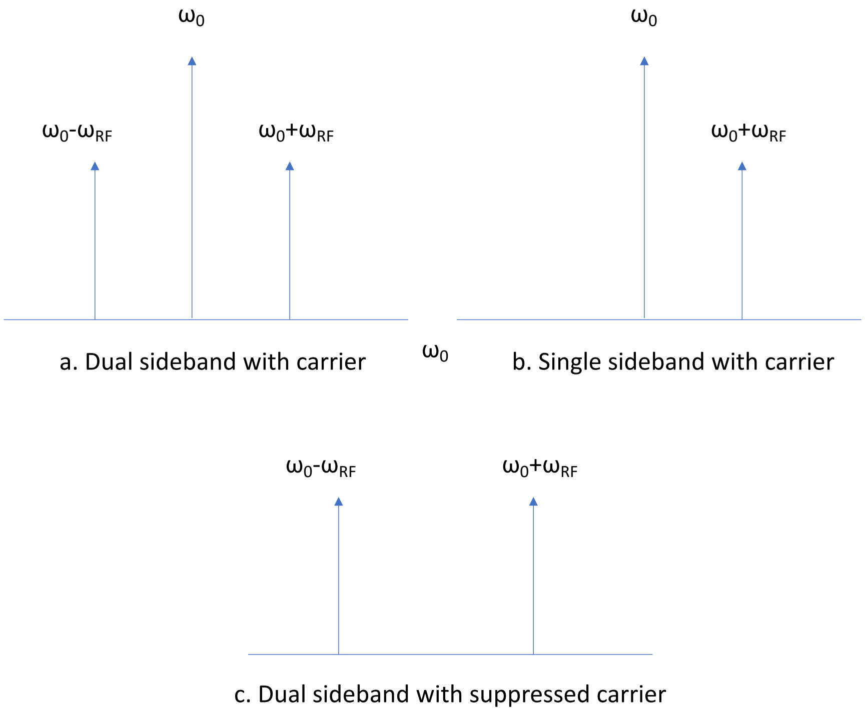

Fig. 2: Different options of intensity modulating an optical carrier with a microwave reference

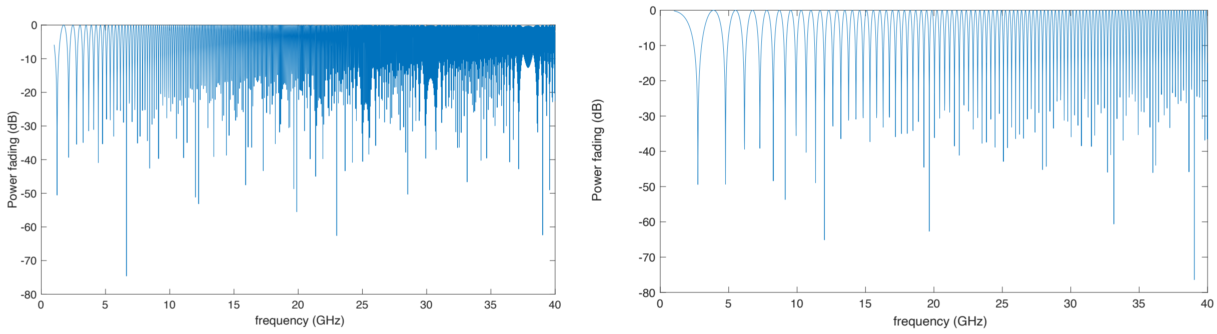

Fig. 3: power fading as a function of RF frequency (f_{RF}) when L=2500 km (left) and L=500 km (right)

Based on fig. 3 one can easily deduce that power fading has different periodicity at different lengths and things become more challenging in terms of preserving the maximum power as the RF frequency increases. However, with the use of a Phase locked loop synthesizer as the one utilized in our experiment, one can set the microwave frequency at the nearest exact maxima for any given L and preserve the stability of the RF frequency in the level of 100s Hz for a long time period (2 ppm PLL reference crystals). The periodicity of maxima for 500 km of transmission is approximately 375 MHz @ 20 GHz and for 2500 km of transmission is approximately 75 MHz @ 20 GHz. Therefore, a general purpose PLL synthesizer can easily lock the frequency at a point of maximum even for very long distances.

Things become much easier when SSB (Fig. 2b) or DSB with suppressed carrier (Fig. 2c) are used. The former requires sophisticated modulation techniques[5], whilst the latter can be easily attained with a simple

[5] Xue, M., Pan, S., & Zhao, Y. (2014). Optical single-sideband modulation based on a dual-drive MZM and a 120 hybrid coupler. Journal of lightwave technology, 32(19), 3317-3323.

modulator by properly adjusting bias and RF modulation depth as explained above. In both cases, only two waves interact at the photodiode, therefore fading is eliminated. The use of DSB with suppressed carrier has a two-fold advantage[1]: on one hand it eliminates power fading, on the other hand it results in microwave frequency doubling, which will enhance the phase resolution of the system according to (4).

The following experimental results show how DSB with suppressed carrier copes with power fading[1]

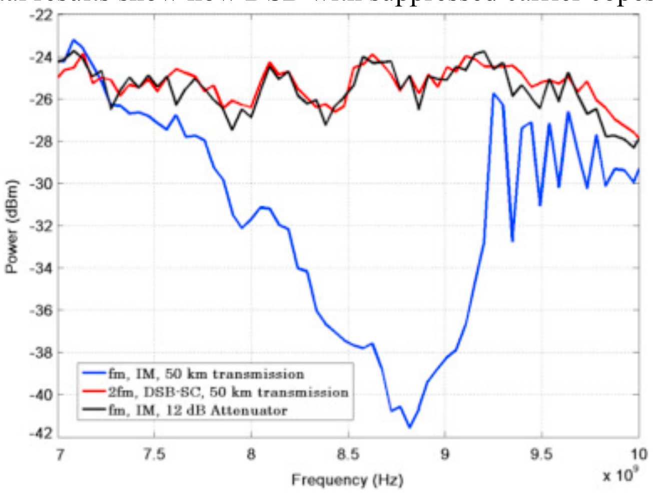

Fig. 4: Power fading as a function of frequency which clearly shows that carrier suppressed DSB is immune to power fading in contrast to DSB with unsuppressed carrier (taken from[1])

B. Processing of measured data

One of the most significant units at the reception state is the one undertaking system monitoring and real-time calibration and processing of the data in order to seamlessly maximize the phase signal. At the reception stage, the microcontroller continuously monitors the output signal and calculates its real-time deviation with respect to the reference voltage level that corresponds to approximately $\pi/2$ phase difference between the microwave generator and the received signal, which maximizes sensitivity. When the real-time error voltage overcomes a specific threshold, a suitable control byte is sent to the HMC642ALC5 microwave phase shifter and the resulting phase transition restores operation close to maximum sensitivity. In addition, a flag byte (outside the range of the output voltage signal) is written by the microcontroller in the output data, indicating the point where phase wrapping occurs. During post-processing, a suitable algorithm localizes the flag bytes in the acquired digital signal and unwraps phase by removing the voltage transition (the control byte) introduced during reception. Fig. 5a shows a snapshot of a raw signal recorded at the receiver where phase corrections are apparent as abrupt vertical transitions. In addition, specific bytes are used as flags to the recorded signal to ensure errorless phase unwrapping. Fig. 5b presents the phase unwrapped signal after post-processing. The arrow depicts the phase variation induced by the earthquake of Crete as an example (October 12, 2021, 09:24:03 UTC, (ML=6.3)) which is visible on the raw data before any post-processing.

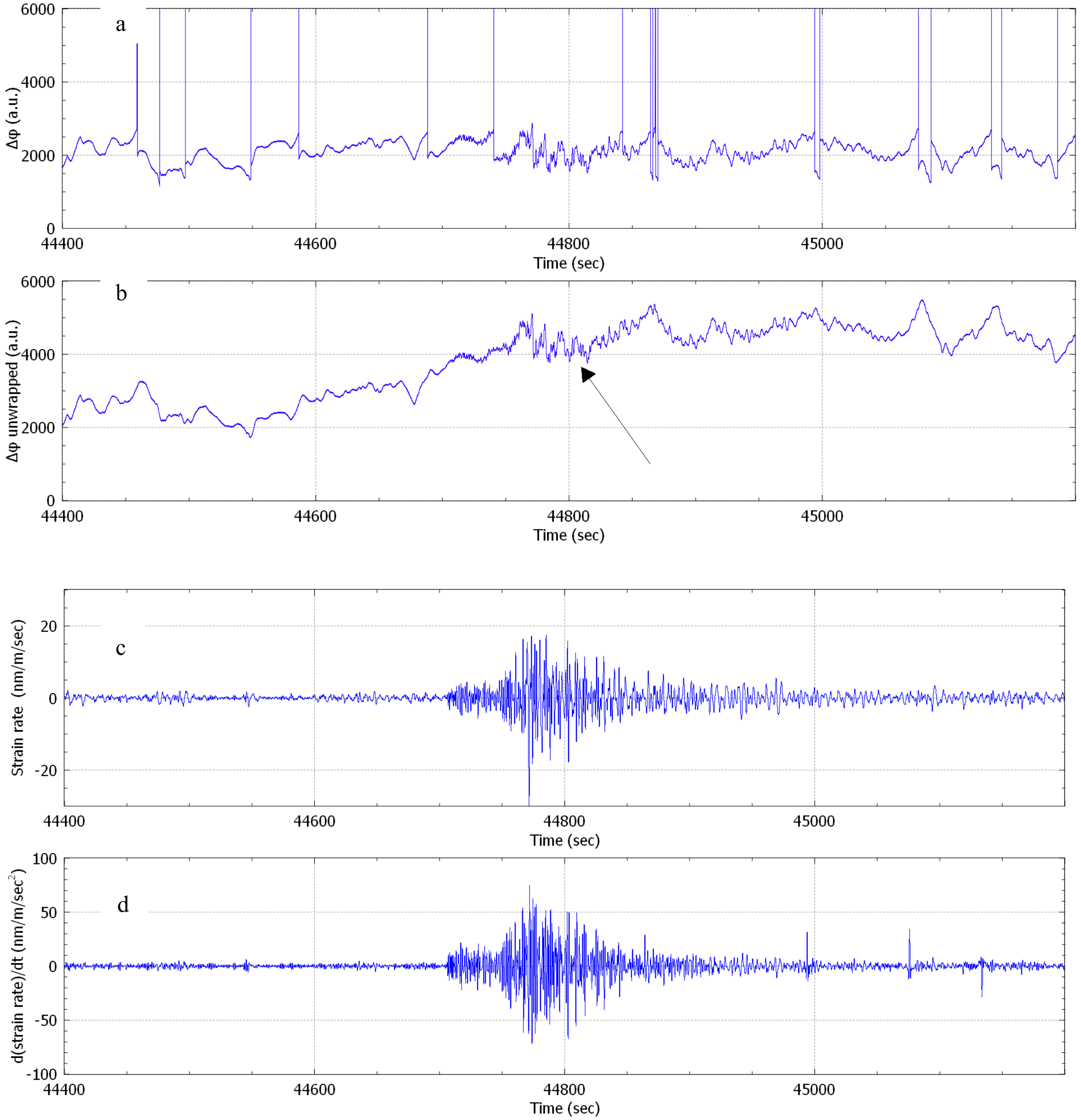

Fig. 5: Step by step processing of phase raw data – the phase shifter provides a continuous correction of the phase shift so as to maximize sensitivity (2000 mV correspond to π/2). a. Raw data, b. data after phase unwrapping, c. strain rate, d. 1st derivative of strain rate.

The output signal which is proportional to phase, is straightforwardly converted to strain by the equation:

$$\varepsilon = \frac{dL}{L} = \frac{d\varphi}{\varphi} = \frac{c d\varphi}{2\pi n_g \xi f_{RF} L},$$

where $\xi \cong 0.78$ is the strain coefficient of the optical fiber due to the photoelastic effect. Further processing converts strain to strain rate $(\frac{d\varepsilon}{dt})$ or acceleration $(d^2\varepsilon/dt^2)$ for comparison with seismometer or accelerometer recordings respectively (see Fig.5c, Fig.5d respectively).

It must be noted that such an active correction mechanism is more difficult to succeed when the phase of the link is interrogated using optical instead of microwave frequencies, due to the rapid changes of phase as a result of environmental effects (optical phase variations exceeding decades of π per second are expected only due to temperature variations). Therefore, despite the fact that microwave frequencies are characterized by lower resolution compared to their optical counterparts, they are also more robust to link noise. This is one of the underlying reasons of MFFI efficacy in providing real-time measurements and consequently event detection in ms scale, despite its use in a noisy terrestrial link positioned in a densely populated area. We anticipate that its performance will be better in less noisy submarine links.

Length of the sensor and its relation to measurement bandwidth

The length of the sensor is not directly related to the useful measurement bandwidth, although it is related to source phase noise (see in methods of the manuscript where fiber loop bandwidth, 4 kHz in our case, is investigated in terms of microwave frequency phase noise). The bandwidth of the final signal is mostly related to the ADC capabilities and the properties of the earthquake. For instance, a distant epicenter will provide signals that reside at lower frequencies (< 5 Hz), whilst a local epicenter will provide earthquakes that acquire higher frequency components (up to 10 Hz). Depending on the information content of the signal, we apply proper filtering in order to highlight the signal and suppress noise.

MFFI – DAS comparison

DAS, as a result of its distributed nature, can estimate strain rate at many locations along the fiber. On the contrary, MFFI provides measurements of the strain which are differentiated to provide strain rate accumulated over the entire link. Therefore, by averaging DAS values we see the agreement with MFFI differentiated signal. Despite the fact that the two systems have analog to digital converters (ADCs) of different sampling rates and resolution, we finally resample the signals at the same rate (50 Hz) and we filter them with the same band-pass filters (0.1 to 1.5 Hz for the case of Crete's earthquake). Their agreement is obvious.

C. Recording of significant earthquake events

We selected more than ten significant earthquakes that took place during the system testing period (July 2021 to February 2022). In this document, we present the recordings from earthquakes with epicenters in Marousi, Ikaria, Kefalonia, Florina, Chalkidiki with epicenters depicted on the map in Figure 2, (left side) in the manuscript. The comparison between MFFI and ATHP data for the selected events are shown below. In all cases a satisfactory agreement is observed, especially noted for the horizontal components. It must be mentioned that MFFI records the seismic wave effect across a 25 km long link, whilst ATHP accelerometric station records the seismic wave at a specific point location. Our next goal is to install accelerometers along the specific link and study the correlation of the accumulated strain provided by the fiber with the superposition of the responses of the in-line accelerometers. This study will provide a better understanding of the strain distribution along the line and will guide us in transforming the MFFI technique to a distributed measurement technique.

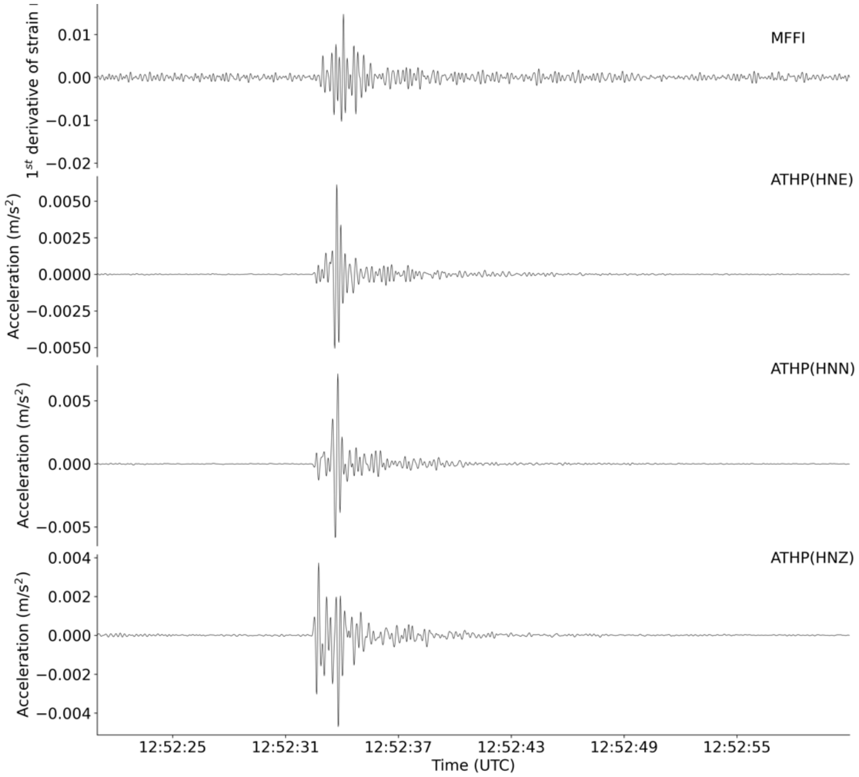

Fig. 6: Comparison of MFFI with ATHP records: Marousi, 25-10-2021, ML=2.8

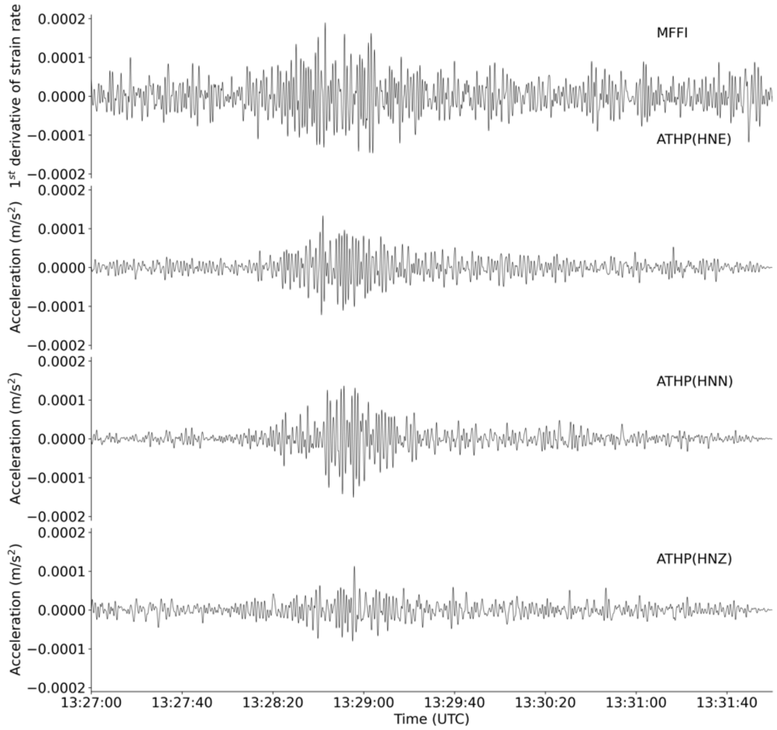

Fig. 7: Comparison of MFFI with ATHP records: Kefalonia, 19-11-2021, ML=4.8

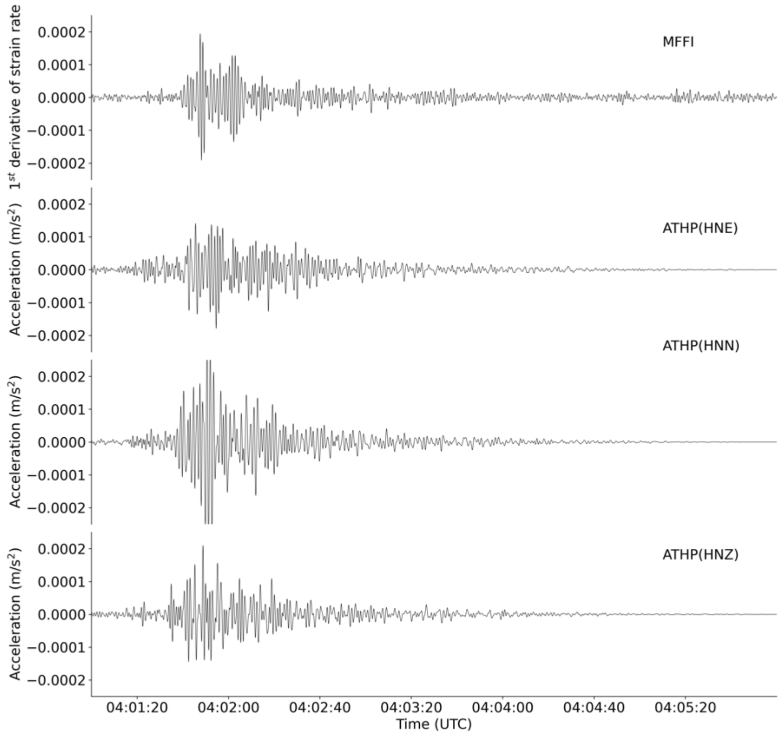

Fig. 8: Comparison of MFFI with ATHP records: Ikaria, 29-11-2021, ML=4.1

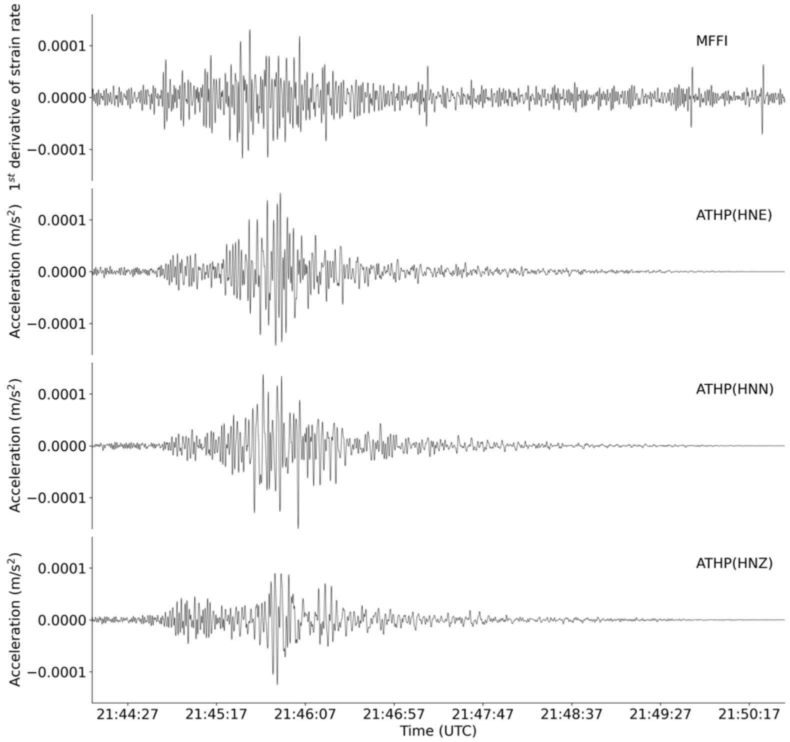

Fig. 9: Comparison of MFFI with ATHP records: Florina, 09-01-2022, ML=5.3

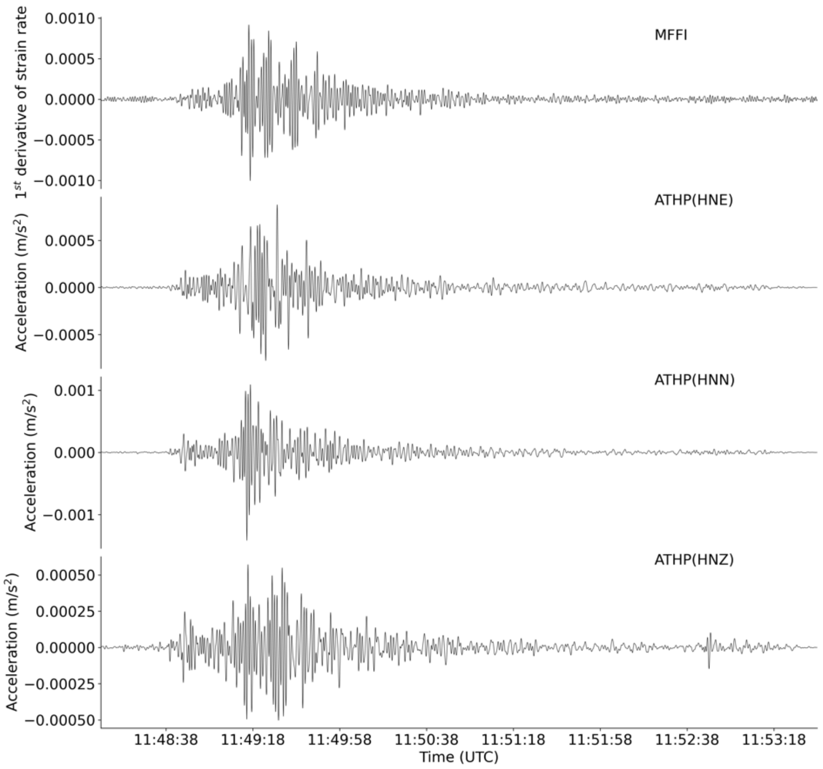

Fig. 10: Comparison of MFFI with ATHP records: Chalkidiki, 16-01-2022, ML=4.4

D. Phase noise of the terrestrial link and MFFI building blocks/stages

Here we present a study of the phase noise of the overall system including the fiber link, the optical and optoelectronic components (optical amplifiers, laser, and photodetector) and the noise of the electronic components (PLL, electrical amplifiers, mixers). We perform the analysis by measuring the one-sided spectral density of phase noise in dBc/Hz as depicted in Fig. 11. In all cases, the output signal is the one digitized by the 10-bit embedded ADC of a low-cost micro-controller (Arduino Uno).

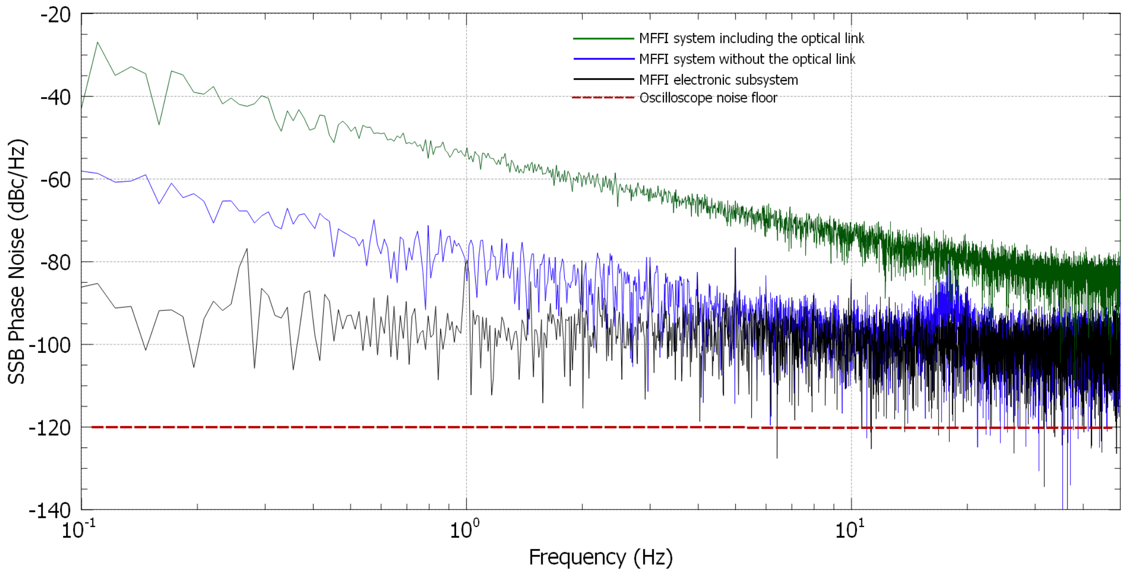

Fig. 11: SSB phase noise in dBc/Hz of MFFI output in various configurations (considered time = 100 sec) – no seismic wave is present in order to characterize the system in terms of the ordinary noise it is subjected to.

Green curve: The phase noise at the output of MFFI is measured when the entire link is included. The high-level spectral density at low frequencies is mostly attributed to link noise (temperature fluctuations, human activity)
Blue curve: The phase noise at the output of MFFI excluding the transmission link which has been replaced by optical attenuators in order to regulate the OSNR at the same level as in the green curve. It is obvious that phase noise has been reduced dramatically, although we observe strong components at low frequencies mostly due to the disturbance of optical components (patch-cords, EDFAs, etc) by mechanical/thermal vibrations inside OTE Academy building. Even at very low frequencies the spectral density of phase noise has been reduced by 20 dB.
Black curve: Here the phase noise is attributed only to the electronic part of the system; it corresponds to the noise floor of the measurement system. Hence the Arduino sampler quantization noise sets the lower limit of phase noise power spectral density approximately to -95 to -100 dBc/Hz.

When we measured the phase noise of the MFFI electronic subsystem with the use of an oscilloscope (Fig. 11 – dashed line) we obtained an improvement of 15-20 dB in the noise floor as depicted, which proves that a better sampling system could further improve MFFI's sensitivity, especially in less noisy environments. This is an improvement that will be applied in the next weeks, by substituting the Arduino 10-bit ADC of 50 Hz with a 14-bit 700 Hz ADC.

E. Localization techniques – converting MFFI to a distributed sensor.

Localization of events is very significant for seismology applications. The following scheme shows the principle of operation already demonstrated in ref. [26] and [51] of the manuscript.

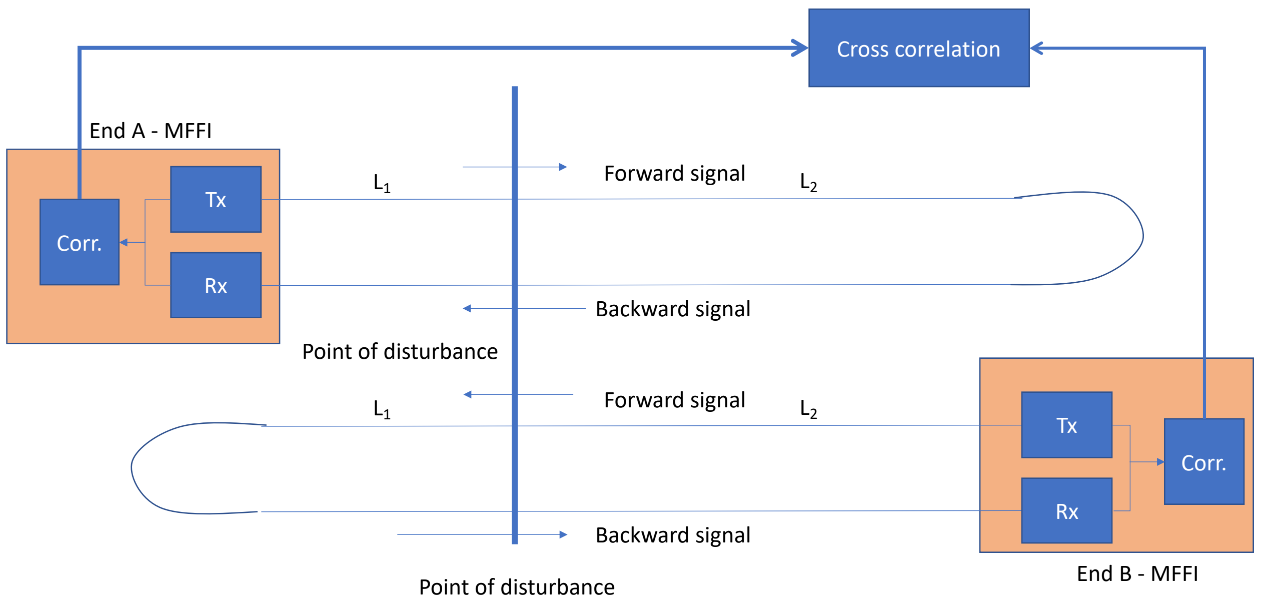

Fig. 12: A potential scheme for transforming MFFI for a lumped sensor to a distributed sensor.

Fig. 12 shows two MFFI interrogators that have been placed at the two ends of a link.

If we rely only on a single MFFI interrogator similarly to [51], we could localize the events as follows: Let us assume that a vibration event affects the fiber at distance L_1 from end A. Then this event will be also repeated after $2L_2$ km as depicted in the figure above ($L_1+L_2=L$ of the link). This length difference will be detected as a time difference in the autocorrelator unit of end A. The time difference will be $\Delta t=2L_2/v_g$. This technique has been evaluated in ref. 51 of the manuscript and showed that hundreds of meters resolution is possible. It is true that DAS can offer resolution in the order of a meter, however this is quite necessary in other types of applications related to intrusion detection, structural health monitoring, etc.

In the field of seismology, the minimum wavelengths that we are dealing with are on the order of several hundred meters or several kilometers. Hence, with a resolution of around 100 m, we would still be able to sample the wavefield completely. Therefore, 100 m resolution is considered adequate.

An extension of this technique is to use simultaneously both A end B MFFI systems. At end B, the same event will provide an autocorrelation signal with $\Delta t=2L_1/v_g$. Thus, a cross correlation of the signals captured by end A and end B will also reveal the position of the event. Marra et al in ref. 26 utilize and demonstrate localization in this manner.

The spatial resolution depends on the ADC sampling rate, that is if we want to target ΔL resolution, then we need an ADC with temporal resolution lower than $\Delta L/v_g$. For 10 meters of spatial resolution we need an ADC of more than 20 MHz bandwidth. The accuracy of the measurement and thus spatial resolution can be enhanced through averaging [26].

The authors also study other techniques towards the goal of transforming MFFI to a distributed sensor (see ref. 52, 53 of the manuscript), which however tackle other mechanisms which are beyond the scope of this work.
